# Does Homeostatic Sleep Pressure Buildup Explain Objective Excessive Daytime Sleepiness in Adults With ADHD? An Exploratory Study

**DOI:** 10.3389/fpsyt.2021.586528

**Published:** 2021-02-19

**Authors:** Stéphanie Bioulac, Patricia Sagaspe, Eléonore Tron, Antoine Benard, Christian Berthomier, Marie Brandewinder, Pierre Philip, Jacques Taillard

**Affiliations:** ^1^CHU Pellegrin, Service Universitaire de Médecine du Sommeil, Bordeaux, France; ^2^Université de Bordeaux, Sommeil, Addiction et Neuropsychiatrie, USR 3413, Bordeaux, France; ^3^CNRS, SANPSY, USR 3413, Bordeaux, France; ^4^CHU Bordeaux, Pôle de santé publique, Service d'information médicale, Clinical Epidemiology Unit (USMR), Bordeaux, France; ^5^PHYSIP, Paris, France

**Keywords:** ADHD (attention deficit and hyperactivity disorder), sleepiness, sleep pressure, maintenance of wakefulness test, phenotype

## Abstract

**Background:** Excessive daytime sleepiness (EDS) is central in Attention deficit hyperactivity disorder (ADHD) but its causes remain unclear. The aim of this study was to explore objective EDS and homeostatic sleep pressure buildup, evaluated by power theta–alpha frequency (PTAF), in drug-free sleepy adults with ADHD and controls.

**Methods:** Participants were placed during a 36-h period of extended wakefulness under constant routine protocol to strictly control sleep time, sleep duration, and circadian zeitgebers.

**Results:** Eight drug-free sleepy patients with ADHD and 7 matched controls were included. The ADHD group had significantly shorter sleep latency on the Maintenance of Wakefulness Test (MWT) throughout extended wakefulness than the control group. There was no significant difference between the groups in PTAF evolution during extended wakefulness and in kinetic sleep pressure buildup, evaluated by the time constant of saturating exponential function.

**Limitations:** The sample was small, so the findings cannot be generalized. Moreover, psychiatric comorbidities and circadian regulation should be taken into account in future studies.

**Conclusion:** In very controlled conditions, mean sleep latency on the MWT during the whole extended wakefulness was significantly shorter in sleepy patients with ADHD than in control subjects. However, the difficulty to remain awake during soporific circumstances observed in these patients with ADHD cannot be explained by changes in the kinetic of sleep pressure buildup.

**Clinical Trials Registration:** www.clinicaltrials.gov/, Identifier: NCT02217371.

## Introduction

Attention deficit hyperactivity disorder (ADHD) is the most common developmental disorder and is characterized by inappropriate levels of inattention, impulsivity, and hyperactivity (1). Follow-up studies have documented the persistence of ADHD into adulthood in 50–65 % of cases (2). In the USA, the prevalence of ADHD in adults has been estimated at 4.4 % (3). ADHD significantly affects major life domains, notably social and occupational functioning. Excessive daytime sleepiness (EDS) is an interesting focus within the context of ADHD in adults as shown in studies using an automatic resting EEG classification of sleepiness (VIGALL, 7 EEG-vigilance stages) who demonstrated unstable arousal regulation in children and adults with ADHD. This arousal instability was characterized by a faster decline to the low EEG-vigilance stages and more fluctuations in their stages of vigilance (4, 5). Moreover, in a study (6) conducted on a population of regularly registered highways drivers: those with ADHD symptoms expressed greater EDS than those who did not have these. This result was confirmed by Ito et al. (7), who showed in a web-based study that the prevalence and severity of EDS in Japanese adults with possible ADHD were higher than in individuals classified as non-ADHD. ADHD is highly associated with primary sleep disorders (8), which could explain the EDS. However, Bioulac et al. (9) found that a significant proportion of adults with ADHD free of primary sleep disorders exhibit objective EDS. Therefore, the cause of objective EDS in ADHD in adults remains elusive and is probably not unique.

Consistent evidence for circadian rhythm disruption in ADHD is now emerging. As described by Coogan and McGowan in their recent systematic review (10), ADHD is associated with evening chronotype and with delayed sleep onset with an objectively measured prevalence of 73–78% in both children and adults with ADHD (11, 12). Delayed sleep onset, or delayed sleep phase syndrome (DSPS), induces a misalignment between circadian time and social time known as social jetlag (13). The social jetlag observed mostly in evening chronotype is associated with sleep restriction, resulting in accumulated EDS over time (13, 14).

Moreover, DSPS may involve changes in the circadian clock drive (Process-C) and/or the sleep homeostatic process (Process-S) (15). Circadian disturbances include an increase in the circadian period, a shorter phase angle, and the dampening of rhythms clock gene expression.

Concerning process-S, we previously demonstrated that evening chronotype feels sleepier during the daytime and that the kinetics of homeostatic sleep pressure build-up differ between morning and evening chronotypes (16). Our hypothesis is that changes in homeostatic sleep pressure build-up could explain EDS in the sleepy adults ADHD subgroup.

To go further than the aforementioned studies, the aim of this study was to explore EDS objectified by MWT and homeostatic sleep pressure in a sleepy ADHD subgroup, and in control subjects for a 36-h period of extended wakefulness under constant routine protocol.

## Methods and Materials

### Population

Patients with ADHD were recruited from the sleep clinic at the University Hospital in Bordeaux (France) according to the *DSM-IVR* criteria (17). The clinical diagnosis of ADHD was validated by standardized instruments. Childhood ADHD and the chronic course of ADHD symptoms from childhood to adulthood were established by a board-certified psychiatrist who carried out a clinical evaluation and administered a semi-structured diagnostic interview (Conners' Adult ADHD Diagnostic Interview for *DSM-IV*, CAADID) (18). The Conners' Adult ADHD Rating Scales (CAARS), observer form, was used. Patients with ADHD were included if they presented a mean sleep latency < 20 min on the Maintenance of Wakefulness Test (MWT) (four MWT trials at 2-h intervals, 10 am, 12 am, 2 pm, 4 pm) (9). According to the American Academy of Sleep Medicine (AASM), the MWT may be used to assess an individual's ability to remain awake when his or her inability to remain awake constitutes a public or personal safety issue (19).

We excluded all patients with any clinically relevant medical or psychiatric condition including current affective or psychotic disorders, substance abuse within 1 year prior to screening, and long-term treatment with benzodiazepine. Comorbid psychiatric disorders were assessed with the Mini International Neuropsychiatric Interview (MINI 5.0.0., *DSM IV*).

All patients were withdrawn from psychostimulant medication for a minimum of 72 h before starting the study.

Healthy control subjects were recruited from the general population and were matched with ADHD patients according to age (±5 years), sex and chronotype, as defined by the score on the Morning Evening Questionnaire (MEQ) (20). We excluded subjects with any psychiatric disorders or any complaint of sleep disorder [reported on the Basic Nordic Sleep Questionnaire (BNSQ)] (21), or an Epworth Sleepiness scale score > 10 (22). ADHD symptoms were ruled out in the control population with the Wender Utah Rating Scale for ADHD (23), ASRS (Adult ADHD Self-Report Scale) (18 items) (24). Controls were included if they presented a mean sleep latency > 36 min on the MWT (four MWT trials at 2-h intervals).

For patients and controls, the presence of nocturnal sleep-disordered breathing (AHI > 10/h) and periodic limb movements (index > 15/h) were ruled out with ambulatory polygraphy. Patients and controls with restless leg syndrome and DSPS were also excluded. Subjects provided written informed consent, and the local ethics committee [consultative committee for the protection of persons participating in biomedical research (CPP Sud-Ouest et Outre-Mer III)] approved the study. The study is registered at Clinicaltrials.gov (NCT02217371).

### Study Design

For 4 days prior to the study, participants were asked to maintain regular bedtimes and wake-up times according to their usual preferences. During this period, compliance was checked by sleep diaries and actimetry. They performed a nocturnal polysomnography (PSG) just before the constant routine to control sleep time and duration. The next morning, 1 h after their preferential wake-up time, all volunteers underwent a 36-h period of extended wakefulness in the constant routine protocol (25). As sleep pressure is generated by the interplay of circadian, homeostatic drives and preferential social timing (16, 26), the night before the constant routine, subjects slept according to their individual and preferential sleep schedule (bedtime and wake time) in order to not modify the kinetic of sleep pressure. Volunteers were kept in a constant semi-recumbent posture in bed and were restricted to very low activity levels, under dim light (<50 lux) and received hourly snacks throughout the day and night.

Sleep pressure was evaluated by theta-alpha (6–9 Hz) band of frontal EEG (27) during the Karolinska Drowsiness Test (4-min eyes-open session, KDT). Frontal power theta–alpha frequency (fPTAF) was calculated (Hanning window) by 4-s epochs after an automatic artifact rejection (ACUTE software, Physip France). Kinetics of sleep pressure buildup were defined by asymptote and time constant assessed by saturating exponential function (27). The first MWT trial began about 1H20 after the subject preferential wake-up time. MWT trials were performed every 4 h (after 1H20 (T1), 5H20 (T2), 9H20 (T3), 13H20 (T4), 17H20 (T5), 21H20 (T6), 25H20 (T7), 29H20 (T8), and 33H20 (T9) of wakefulness). Subjects followed classical MWT instructions (28). Trials were ended after 40 min if no sleep occurred or after three consecutive epochs of stage 1 sleep or after one epoch of any other stage of sleep. Sleep latency was defined as the first epoch of any stage of sleep.

During PSG, KDT and MWT, EEG, EOG, and EMG were recorded on a Brainbox EEG-1042 Amplifier with coherence software (Natus, France). All electrophysiological signals were digitized at a sampling rate of 256 Hz. KDT and MWT were repeated every 4 h.

## Statistical Analysis

Actimetry and PSG measures were compared between ADHD patients and controls using Wilcoxon rank tests. Repeated outcomes (MWT and Process S fPTAF) as well as the constant and the asymptote of the fPTAF were compared between groups using linear mixed effects models adjusted for age, gender, and Hörne and Ostberg score, and taking into account an interaction between group and time. The analyses were conducted on available data, using SAS software v9.4 (SAS Institute Inc., Cary, NC, USA). *p*-Values <0.05 were considered statistically significant.

## Results

### Population

Eight ADHD patients and 9 control participants were recruited. Two control participants withdrew from the experiment during the constant routine protocol. The sample thus consisted of 15 subjects: 8 ADHD patients (mean age = 39.8 ± 11 years, range 21–53 years, BMI = 24.6 ± 4.7, 2 males) and 7 control participants (mean age = 42.6 ± 9.1, range 25–53, BMI = 24.0 ± 3.8, 1 male). The mean MEQ score in ADHD patients was 56.8 ± 12.7, demonstrating that sleepy ADHD patients were mostly intermediate chronotypes. Only one sleepy ADHD patient was classified as evening chronotype.

Among the 8 patients with ADHD included, 50% presented a psychiatric comorbidity (25% had comorbid anxiety disorder (past or present) and 50% had a history of mood disorders but no current mood disorder). Fifty percent presented with ADHD of the mixed subtype and 50% with the inattentive subtype. Patients with ADHD presented the following scores on the CAARS (58.4 ± 15.7) and the Brown questionnaire (69.8 ± 26.3).

Table 1 shows the demographic and clinical characteristics of ADHD patients and controls.

**Table 1:** Demographic and clinical characteristics (Mean ± SD) of sleepy ADHD patients and control participants at inclusion.

	**ADHD patients** **(*n* = 8)**	**Controls** **(*n* = 7)**
**Demographic and clinical characteristics**		
Age (years)	39.8 ± 11.0	42.6 ± 9.1
Gender (% females)	75%	85.7%
BMI (kg/m^2^)	24.6 ± 4.7	24.0 ± 3.8
Mean sleep latency on MWT (min)	13.9 ± 4.5	38.9 ± 2.0
**Self-reported questionnaires**		
MEQ score	56.8 ± 12.7	57.9 ± 2.8
ESS score	16.9 ± 2.4	3.4 ± 2.5
WURS score	55.5 ± 9.4	10.6 ± 7.6

*ADHD, attention deficit/hyperactivity disorder; BMI, body mass index; MWT, maintenance of wakefulness test; MEQ, morningness–eveningness questionnaire; ESS, Epworth Sleepiness scale; WURS, Wender Utah Rating Scale*.

### Actimetry

There was no significant difference between patients with ADHD and controls in total sleep time for the four nights before the protocol (Night 1: 422.6 ± 176.0 min vs. 428.0 ± 36.2 min, *p* =0.41; Night 2: 453.3 ± 128.2 min vs. 437.0 ± 52.2 min, *p* = 0.73; Night 3: 419.8 ± 63.9 min vs. 385.3 ± 73.6 min, *p* = 0.39; Night 4: 429.3 ±110.5 vs. 415.7± 40.2, *p* = 0.73).

### Polysomnography

There was no significant difference between patients with ADHD and controls in total sleep time on PSG the night before the protocol: 404.8 (50.0) min vs. 392.9 (62.0) *p* = 0.69.

### MWT

**Figure 1:**
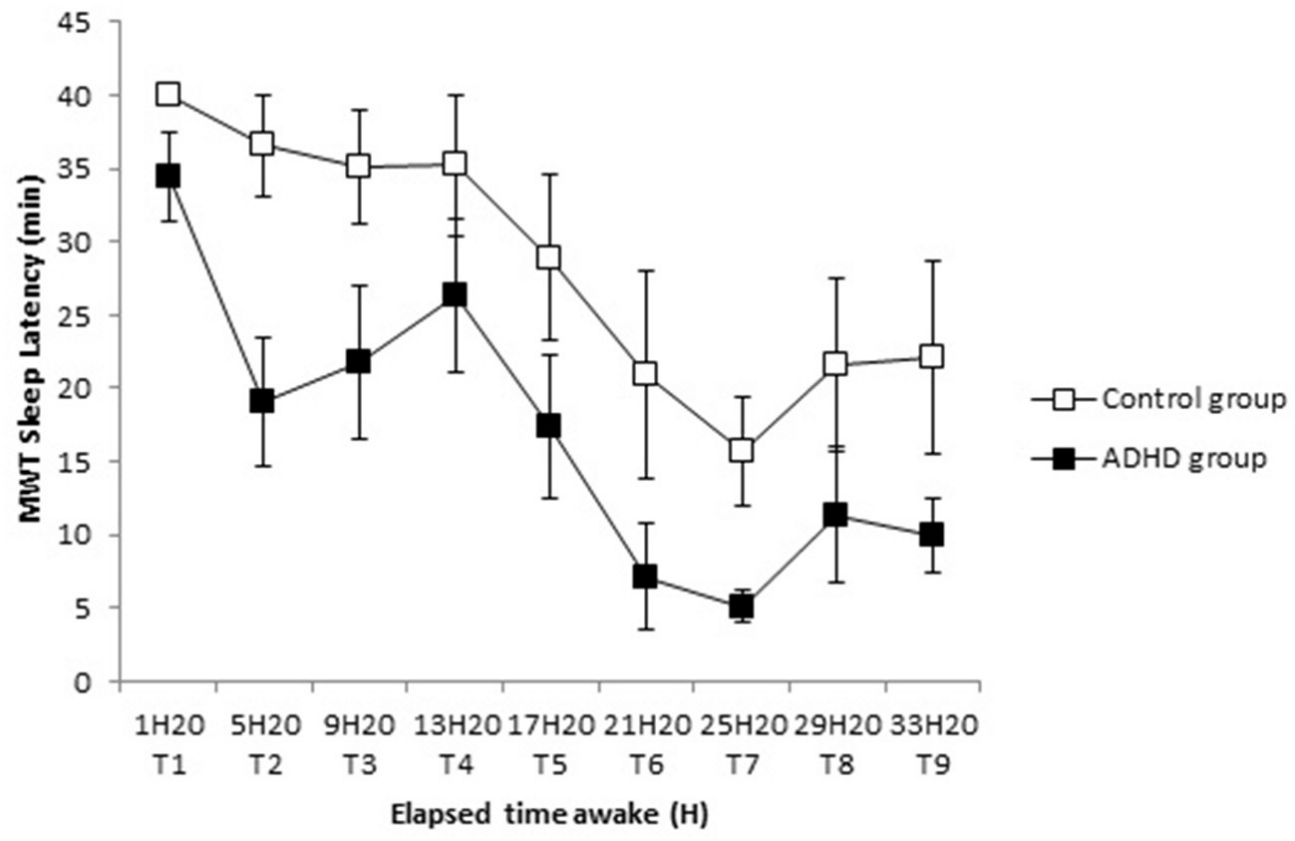
Time course of sleep latency on MWT (Mean ± SE) in sleepy ADHD group and control group during 36-h of sustained wakefulness from T1 to T9.

The ADHD group had a shorter sleep latency on the MWT than the control group at T1 [34.4 ± 8.7 min vs. 40.0 ± 0.0 min (*p* = 0.013)]. This significantly shorter sleep latency on the MWT persists over time, but remains stable over the eight measures (*p* = 0.94) (Figure 1).

### Process S

**Figure 2:**
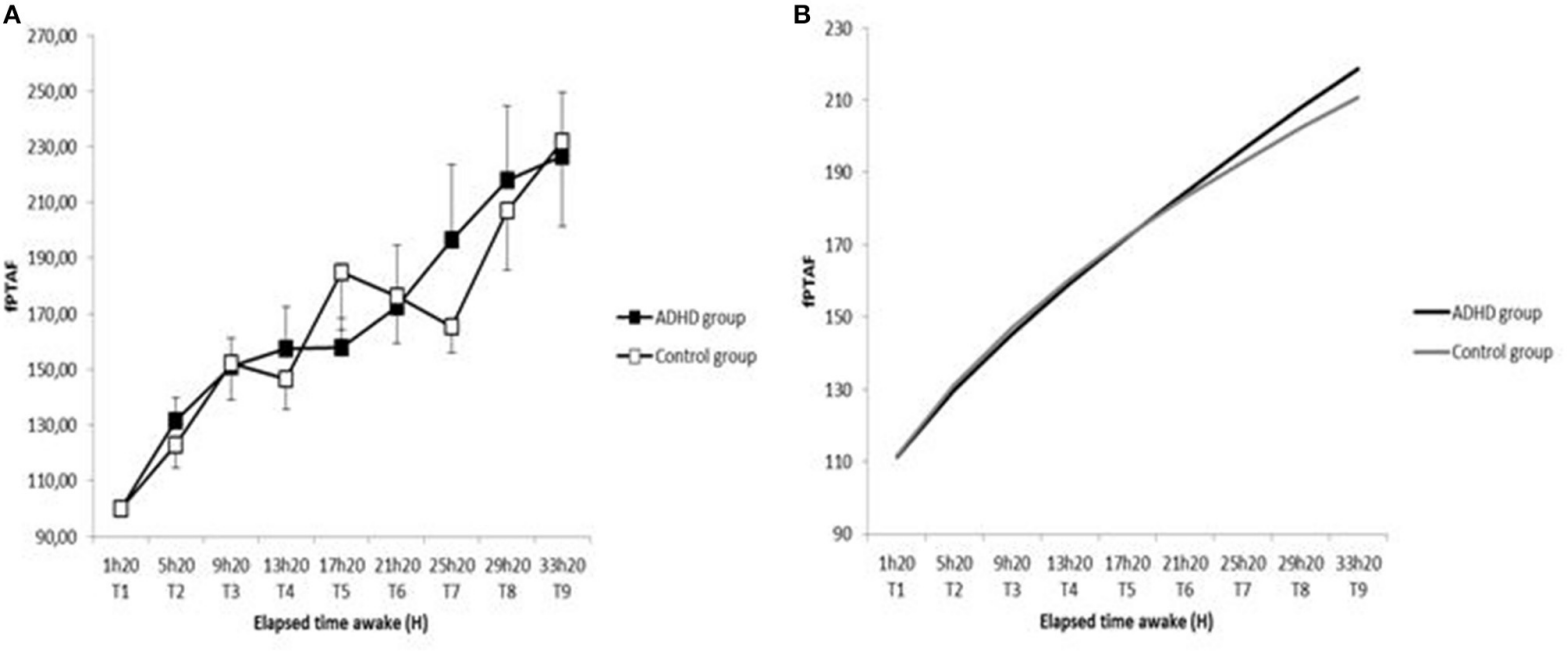
Time course of fPTAF in sleepy ADHD group and control group during 36-h of sustained wakefulness from T1 to T9 **(A)** and sleep pressure buildup in sleepy ADHD and controls assessed by saturating exponential function **(B)**.

There was no significant difference in fPTAF between the groups, whether at T1 (126.5 ± 173.2 vs. 95.9 ± 101.4 (*p* = 0.80), for ADHD patients and controls, respectively) or regarding the evolution of the eight other measures (*p* = 0.37) (Figure 2).

Regarding kinetics of sleep pressure buildup, there was no significant difference between the groups in mean time constant (33,043.4 ± 68,519.6 h for ADHD patients and 126,303.9 ± 187,901.4 h for controls; *p* = 0.12), nor in mean asymptote (112,874.9 ± 206,194.4 for TDAH and 534,873.4 ± 10,004,871.6 for controls; *p* = 0.18).

## Discussion

This study is the first to explore the impact of sustained wakefulness on objective EDS in drug-free sleepy adults with ADHD during a 36-h period under constant routine. In highly controlled conditions (sleep time, sleep duration and circadian zeitgebers), mean sleep latency on MWT remained significantly shorter in sleepy patients with ADHD than in control participants. Indeed, at the beginning of the protocol (first MWT), sleepy patients with ADHD displayed a significantly shorter mean sleep latency on the MWT than control participants and this difference persists throughout the whole constant routine remaining stable. Contrary to our original hypothesis, difficulty to remain awake during soporific circumstances cannot also be explained by a change in homeostatic sleep pressure since the kinetics of sleep pressure build-up did not differ between ADHD patients and control subjects.

This kind of protocol permits to test fundamental hypothesis about the origin of sleepiness in this subgroup of patients with ADHD. EDS is sometimes caused by insufficient sleep, a delay in the sleep phase (29) or by misalignment between circadian time and social time (social jetlag) (13). As all participants were matched in chronotype and were asked to follow regular and preferred sleep-wake schedules and habitual sleep duration (compliance verified by actimetry) during the 4 days before the constant routine, the EDS observed in our sleepy patients with ADHD cannot have been artificially induced by sleep restriction or social jetlag. Importantly, total sleep time did not differ between the two groups before the constant routine on actimetry and PSG.

These findings suggest that EDS in ADHD may have a central origin, leading to a state of hypo-arousal state, as in narcolepsy. Miano et al. identified five specific sleep phenotypes in ADHD children (30) including one called “narcolepsy-like phenotype” that could correspond to our sleepy ADHD subjects. This hypothesis is supported by the recent work of Diaz-Roman et al. (31), who found shorter REM latency and higher levels of EDS in children with ADHD than in control children. These data might indicate early signs or shared symptoms of narcolepsy in these children with ADHD. In support of a continuum between ADHD and central hypersomnia, Lopez et al. (32) found a high frequency of ADHD and ADHD-like symptoms in patients with central hypersomnia, explaining high levels of EDS and hypersomnolence in adults with ADHD. In a new model of sleep/wake regulation, Fulcher et al. (33) extended the model of Phillips and Robinson (34) by demonstrating that the dynamics of sleep and wake may be controlled not only by circadian rhythms and homeostatic drive but also by orexin levels. The model posits that a reduction in orexin levels leads to reduced daytime arousal without altering any other drives, as observed in our sleepy ADHD subgroup.

Moreover, we cannot exclude the effect of circadian changes associated with ADHD especially the phase delay of circadian phase (11, 35) on EDS even if the patients with ADHD included in this study were classified mostly in intermediate chronotype. Although it is still not certain whether circadian amplitude is impaired in ADHD patients, further studies will have to fully explore changes in circadian phase, circadian period, circadian entrainment, and/or polymorphisms in genes controlling the circadian drive.

This study has several limitations. First, the sample was small given the heaviness of the protocol, so the findings cannot be generalized. Consequently, we could not take *DSM-IV* subtypes into account. Second, our patients had psychiatric comorbidities, half of them suffering from another psychiatric disorder, as expected. A limit of our study is that we did not control for stressful life events which typically can be associated with reduced sleep causing daytime sleepiness. Therefore, the EDS in our patients with ADHD may have been influenced by psychiatric comorbidities. Moreover, we cannot exclude the effect of circadian changes associated with ADHD especially the phase delay of circadian phase on EDS even if the patients with ADHD included in this study were classified mostly in intermediate chronotype. Although it is still not certain whether circadian amplitude is impaired in ADHD patients (10), further studies will have to fully explore changes in circadian phase, circadian period, circadian entrainment, and/or polymorphisms in genes controlling the circadian drive. Finally, we cannot totally exclude an after effect over 72 h due to withdrawal-related hypersomnia symptoms (36). However, in the literature, most of ADHD-related studies commonly stopped psychostimulants treatment for a period of 48–72 h before the protocol (37, 38). Further studies will have to control this limiting factor.

In conclusion, this study conducted in highly controlled conditions revealed a significant and stable difference in mean sleep latency on the MWT between drug-free sleepy patients with ADHD and control subjects. The difference cannot be explained by specific changes in the kinetics of sleep pressure buildup with ADHD. EDS is a key objective biomarker to better evaluate ADHD patients. The clinical implications are that personalized pharmacologic treatment with wakefulness-promoting drugs could improve cognition and behaviors in sleepy ADHD patients.

## Data Availability Statement

The raw data supporting the conclusions of this article will be made available by the authors, without undue reservation.

## Ethics Statement

The studies involving human participants were reviewed and approved by Consultative Committee for the protection of persons participating in biomedical research [CPP Sud-Ouest et Outre-Mer III—Clinical Trials Registration: NCT02217371]. The patients/participants provided their written informed consent to participate in this study.

## Author Contributions

SB, PS, PP, and JT: designed this study. SB, PS, JT, ET, AB, CB, and MB: coordinated the data collection and enrolment of participants. SB, PS, and JT drafted the manuscript which was added to and modified by AB, ET, CB, MB, and PP. All authors read and approved the final manuscript.

## Conflict of Interest

The authors declare that the research was conducted in the absence of any commercial or financial relationships that could be construed as a potential conflict of interest.
